# Dynamics of NKT-Cell Responses to Chlamydial Infection

**DOI:** 10.3389/fimmu.2015.00233

**Published:** 2015-05-15

**Authors:** Sudhanshu Shekhar, Antony George Joyee, Xi Yang

**Affiliations:** ^1^Department of Medical Microbiology, Faculty of Medicine, University of Manitoba, Winnipeg, MB, Canada; ^2^Department of Immunology, Faculty of Medicine, University of Manitoba, Winnipeg, MB, Canada

**Keywords:** CD1d restriction, NKT cells, dendritic cells, *Chlamydia*

## Abstract

Natural killer T (NKT) cells have gained great attention owing to their critical functional roles in immunity to various pathogens. In this review, we provide an overview of the current knowledge on the role of NKT cells in host defense against and pathogenesis due to *Chlamydia*, which is an intracellular bacterial pathogen that poses a threat to the public health worldwide. Accumulating evidence has demonstrated that NKT cells, particularly invariant NKT (iNKT) cells, play a crucial role in host defense against chlamydial infections, especially in *C. pneumoniae* infection. iNKT cells can promote type-1 protective responses to *C. pneumoniae* by inducing enhanced production of IL-12 by dendritic cells (DCs), in particular CD8α+ DCs, which promote the differentiation of naive T cells into protective IFN-γ-producing Th1/Tc1 type CD4+/CD8+ T cells. This iNKT-cell-mediated modulation of DC function is largely dependent upon CD40–CD40L interaction, IFN-γ production, and cell-to-cell contact. In addition, iNKT cells modulate the function of natural killer cells. NKT cells may be also involved in the pathogenesis of some chlamydial diseases by inducing different patterns of cytokine production. A better understanding of NKT-cell biology will enable us to rationally design prophylactic and therapeutic tools to combat infectious diseases.

## Introduction

Natural killer T (NKT) cells represent a unique population of innate lymphocytes that express the natural killer (NK)- and T-cell markers, such as NK1.1 and αβ T-cell receptor (TCR) (1). NKT cells are divided into two subsets, type I or invariant NKT (iNKT) and type II NKT cells, depending upon their TCR forms and cognate ligands. iNKT cells possess the invariant αβ TCR (iTCR) that recognizes glycolipid and lipid antigens presented to them by CD1d molecules. iNKT cells are the most widely studied subset of NKT cells. Activation of iNKT cells leads to the rapid production of Th1, Th2, and Th17 cytokines and chemokines (2). On the other hand, type II NKT cells do not express the iTCR and are reactive to sulfatides (1). Because of their distinct immunologic characteristics and crucial functions in host immune responses to different pathogens, NKT cells have gained much attention in recent years (3). In this review, we focus on the functional role of NKT cells, especially iNKT cells, in conferring T-cell immunity against chlamydiae, which are obligate intracellular bacteria that cause a range of human diseases worldwide (4). In particular, we describe how iNKT cells bridge innate and adaptive immunity by modulating the function of dendritic cells (DCs) during chlamydial infection. These findings present a rational basis for developing effective prophylactics and therapeutics against infectious diseases.

## Chlamydial Infections and Their Pathophysiology

*Chlamydia* has a biphasic life cycle, consisting of two distinct forms, elementary body (EB) and reticulate body (RB). EB is an extracellular and metabolically inactive, but stable form, which is responsible for dissemination of infection from one person to another. On the contrary, RB is an intracellular and metabolically active form (5). EBs attach and enter the epithelial cells through endocytosis. Following their entry into the cell, the EBs undergo germination to give rise to RBs. The RBs so formed multiply by binary fission in enlarging vacuoles called inclusion bodies. As the inclusion body expands following maturation, the RBs re-differentiate into EBs, which are released by the cells to infect more cells (6).

Chlamydial species belong to the taxonomic family Chlamydiaceae. Using 16s and 23s rRNA sequencing, the family Chlamydiaceae has divided into two genera and nine species (7). Out of these nine chlamydial species, *C. trachomatis* and *C. pneumoniae* (also called as *Chlamydophila pneumoniae*) are clinically significant species that cause a variety of human diseases. *C. trachomatis* has three human serovars, including serovars A–C, D–K, and L1–L2. Serovars D–K of *C. trachomatis* are the most common cause of bacterial sexually transmitted diseases (STDs), but can also cause neonatal pneumonia and conjunctivitis (4, 8–11). They cause 90 million cases of STDs each year across the globe, with approximately 3 million cases alone in the United States (11). The clinical manifestations of chlamydial genital infection in women include urethritis, cervicitis, upper genital tract infection, and perihepatitis. If untreated, infected women can develop pelvic inflammatory disease, which have serious consequences, such as infertility, ectopic pregnancy, and abortion. In men, *C. trachomatis* infection can cause urethritis, epididymitis, seminal vesiculitis, and prostatitis (8, 9). It is notable here that majority of infected people are asymptomatic and only about 20% of infected women and 30% of men show clinical signs of chlamydial infection and so are subjected to antibiotic treatment. A range of broad spectrum antibiotics such as erythromycin and tetracycline are effective against *Chlamydia*, although an accurate and timely diagnosis of chlamydial infections presents a challenge to the clinician due to their diverse clinical manifestations (4, 12). Serovars L1–L2 of *C. trachomatis* cause lymphogranuloma venereum, which is a venereal disease with lesions in genital tissues, particularly the tissue-draining lymphatics and lymph nodes. These genital tract chlamydial infection increases the chance of women to be infected with human immunodeficiency virus and human papilloma virus-induced cervical neoplasia (13, 14). Apart from genital tract infection, serovars A–C of *C. trachomatis* cause trachoma, which is the leading cause of infectious blindness worldwide that affects about 84 million people with active disease. Pathologic lesions in trachoma include the development of follicles and inflamed conjunctivae that lead to cloudy and vascularized cornea, trichiasis, corneal ulcer, and blindness. Transmission of *C. trachomatis* for trachoma takes place by contaminated fingers or fomites or through placenta in infected mothers (15, 16). On the other hand, *C. pneumoniae* causes a variety of respiratory diseases, including sinusitis, pharyngitis, bronchitis, and community-acquired pneumonia that are common throughout the world (17). A higher prevalence of chlamydial infection is however noted in third world countries compared to the developed ones. Humans are the only known reservoir for *C. pneumoniae*. In recent years, there are various reports based on epidemiological, immunological, and pharmacological studies that indicate an association of *C. pneumoniae* infection with cardiovascular and neurodegenerative diseases, such as atherosclerosis, Alzheimer’s disease, and multiple sclerosis (18–20). Despite considerable efforts, it still remains a challenge to develop a safe and effective chlamydial vaccine due to inadequate knowledge of protective immunity and immunopathology of chlamydial infections. This is important because of the fact that the immune responses also contribute to the pathogenesis of chlamydial diseases (21). An effective vaccine strategy therefore requires the identification of antigens/adjuvants, which evoke protective but not pathologic immune responses.

Coexistence of *Chlamydia* and its host imposes an evolutionary pressure on both of them. The host’s immune system has developed to defend the body from chlamydial infections, whereas *chlamydiae* are equipped with various evasion mechanisms to escape the host’s immune system. Pathogenesis of chlamydial diseases is the result of this host–pathogen interaction. Chlamydial infection leads to the activation of mucosal epithelial cells. Activation of epithelial cells induces secretion of multiple cytokines and chemokines, such as IL-1, TNF-α, IL-8, GM-CSF, and IL-6, which cause infiltration of immune cells at the primary site of infection. These immune cells include, but not limited to, neutrophils, monocytes, NK cells, and T cells. Infected epithelial cells and neutrophils secrete potent proteolytic enzymes like elastase and MMPs to cause tissue damage (22–24). Persistence of chlamydial infection can lead to the continuous release of proinflammatory cytokines from the epithelial cells which results in tissue damage. On the other hand, immune responses have also been held responsible for the tissue damage. Although IFN-γ+ CD4 T cells induce immunity to chlamydial infection, they might have detrimental effects on the primary site of infection resulting in collateral damage (25). CD4 T cells producing IL-4 can elicit immunopathology via suppression of protective responses (26). Autoreactive T cells specific for *Chlamydia* and host proteins such as heat-shock protein 60 have also been described, although the mechanism of their development can be assigned to the phenomenon of molecular mimicry (27). A reduced pathology in IL-knockout (KO) mice, compared to the wild-type (WT), during *Chlamydia*-infected mice suggests a detrimental role for IL-10 in this infection model (28, 29). Therefore, overt responses by immune cells can culminate into pathology during chlamydial infection.

## Anti-Chlamydial Host Immunity

Many studies in animal models and clinical settings have demonstrated that T cells play a crucial role in control of chlamydial infections. In genital infection of *C. trachomatis*, CD4+, but not CD8+, T cells are indispensable for resolution of primary as well as secondary infections (30–33). Similar function for CD4+ T cells has been described in *C. muridarum*, a mouse biovar of *C. trachomatis*, and lung and genital tract infection (34). Although both CD4+ and CD8+ T cells contribute to immunity to *C. pneumoniae* lung infection, the predominant role is played by CD8+ T cells (35). The type of T-cell immunity has a profound effect on whether the infection is contained or culminates into pathology. Th1/Tc1 responses characterized by IFN-γ production by CD4+/CD8+ T cells are the major form of protective immunity (34). It is also shown that IL-17/Th17, in cooperation with Th1, responses exert anti-chlamydial adaptive immunity, especially in lung infections (36–38). In contrast, Th2 immunity characterized by secretion of cytokines such as IL-4, IL-5, and IL-13 are more associated with inflammatory and pathologic changes (34). IL-10, a Th2 and immunoregulatory cytokine, has also been annexed with pathologic responses (28). Therefore, promotion of Th1/Tc1 and, in certain conditions, Th17 responses are more likely beneficial for resolution of chlamydial infection, whilst the increase of Th2 responses culminates into pathology.

## Activation of iNKT Cells

Activation of iNKT cells is achieved through two mechanisms; CD1d-dependent and CD1d-independent. In CD1d-independent mechanism, iNKT cells are activated without involvement of CD1d molecules. This type of activation is mediated through innate or inflammatory stimuli irrespective of the presence of foreign microbial antigens, possibly in conjunction with self-glycolipid antigen recognition (39). Recent data further illustrate that the innate stimuli such as cytokines appear to be the predominant means of iNKT-cell activation, even with bacteria that carry iNKT cell agonists (40). On the other hand, in CD1d-dependent mechanism, antigens are presented by CD1d molecules expressed on antigen-presenting cells (APCs) such as DCs for interaction with iTCR that possesses a conformation that is able to recognize glycolipid and lipid antigens processed and presented to them by CD1d molecules (41, 42). Interaction between iTCR and its cognate ligand leads to the activation of iNKT cells, as evidenced by massive production of a variety of cytokines, such as Th1 (IFN-γ), Th2 (IL-4), and Th17 (IL-17) cytokines and chemokines (43). The biochemical and physiological nature of iNKT-cell-specific ligands has been deciphered by many recent studies. Kawano et al. for the first time identified a lipid antigen specific for iNKT TCR, α-galactosylceramide (α-GalCer), which was originally isolated from a marine sponge, *Agelasmauritianus* (44). Since α-GalCer is a potent ligand for iNKT-cell activation and has been instrumental in understanding the biological properties of iNKT cells, it is referred to as a prototypic antigen for these cells. The ability to activate iNKT cells is however not limited to α-GalCer. A variety of microbial antigens have also been shown to activate iNKT cells, such as α-glucuronosylceramide from *Sphingomonas* species (45–47), α-galactosyldiacylglycerol from *Borrelia burgdorferi* (48), and phosphatidylinositol-mannosidase from *Mycobacterium bovis* BCG (49, 50). In an attempt to identify chlamydial lipid antigens which active iNKT cells, we tested a previously reported glycolipid exoantigen from *C. muridarum* (GLXA) for activation of iNKT cells (51, 52). We found that intravenous injection of GLXA into WT mice led to an enhanced production of IFN-γ and IL-4 in mouse sera, which was not seen in Jα18-KO mice that lack iNKT cells only. Following GLXA treatment, iNKT cells underwent activation and produced IFN-γ and IL-4 (52). These findings suggest that chlamydial GLXA acts as a specific ligand for iNKT-cell activation. In line with these findings, Jiang et al., using APC-free culture system, have shown that both iNKT as well as type II NKT-cell hybridomas were activated when cultured with ultraviolet-killed *C. muridarum* (53). It is known that iNKT and type II NKT cells are activated by different ligands. For example, lipid and glycolipids are ligands for iNKT-cell activation, whereas hydrophobic antigens such as sulfatides induce specific activation of type II NKT but not iNKT cells. Since *C. muridarum* activated iNKT and type II NKT cell hybridomas, it is likely that there are different chlamydial antigens for activating these cell types (53). Therefore, further studies on identification and purification of different chlamydial antigens for iNKT- and type II NKT-cell activation may be crucial for anti-chlamydial vaccine development. Based on the current data, it appears reasonable to conclude that iTCR is involved in activation of iNKT cells through interaction with chlamydial antigens.

## iNKT Cells in Protective Immunity Against Chlamydial Infection

Recent studies in mice have provided significant evidence on the role of NKT cells in protective immunity to various infections, including chlamydial infections (54, 55). Activation of iNKT cells by injection of α-GalCer in mice mounted a strong protective immunity to intranasal *C. pneumoniae*, intra-articular *C. trachomatis*, and intravaginal *C. muridarum* infection (54, 56, 57). In these studies, BALB/c mice were used for intra-articular *C. trachomatis* and vaginal *C. muridarum* infection, and C57BL/6 mice for intranasal *C. pneumoniae* infection; however, the outcomes of the infections were similar following α-GalCer treatment (54, 56, 57). To better understand the protective function of NKT cells *in vivo* during chlamydial infections, we and other groups used various experimental approaches, including transgenic/KO mice. During *C. trachomatis* intra-articular infection, CD1d-KO mice, which lack both iNKT and type II NKT cells, experienced enhanced pathology and higher bacterial burden compared to the WT mice, indicating a protective role for NKT cells in this infection model (57). To directly examine the contribution of iNKT cells in host defense against *C. pneumoniae* infection, we infected Jα18-KO mice through intranasal route (54). Upon infection challenge, more severe body weight loss, pathological changes, and higher organism growth were observed in Jα18-KO mice than in the WT mice (54), which suggested a protective effect of iNKT-cell activation on *C. pneumoniae* infection. We further assessed the impact of iNKT cells on T cells in the context of cytokine response to *C. pneumoniae* infection (54). Intracellular cytokine analysis demonstrated that the WT mice, compared to the KO, displayed a robust type-1 CD4 and CD8 T cell response, characterized by IFN-γ production in *C. pneumoniae* infection. Furthermore, CD4 T cells of Jα18-KO mice reflected an enhanced Th2 (IL-4) response than those from the WT mice (54). Thus, iNKT cells contribute to the development of protective Th1/Tc1 responses against *C. pneumoniae* infection.

## Bridging Innate and Adaptive Immunity

**Figure 1:**
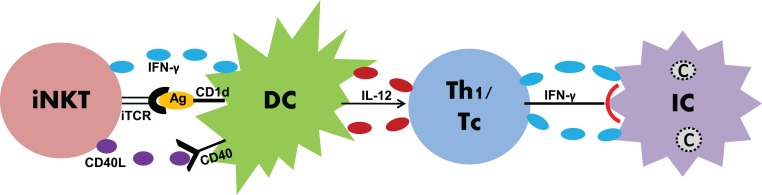
**Induction of anti-chlamydial T-cell responses by iNKT cells through DC modulation**. During chlamydial infections, iNKT cells induce DC maturation through IFN-γ, CD40–CD40L binding, iTCR–Ag interaction, and cell-to-cell contact. Once matured, DCs induce enhanced production of IL-12 that skews Th1/Tc responses. Th1/Tc responses characterized by IFN-γ production lead to the clearance of intracellular Chlamydiae.

It is becoming clearer that innate and adaptive immune systems do not work in isolation, but rather interact with each other to give rise to an optimal immune response against infections. A significant example in this context is the case of innate lymphocytes that have been shown to bridge innate and adaptive immunity by modulating DCs (58). To study the impact of iNKT cells on DC function, α-GalCer as a model antigen has been widely used. Whether this is true in case of real infections has been addressed by some recent studies (59–63). Our recent studies using a mouse model of *C. pneumoniae* infection have done an in-depth analysis of the impact of iNKT cells on DC function for the elicitation of T-cell immunity in a real infection setting (62, 63) (Figure 1). Adoptive transfer of DCs isolated from the spleens of *C. pneumoniae*-infected Jα18-KO, in contrast to the WT, mice promoted infection and pathology in naïve recipient mice upon challenge with chlamydial infection (62), suggesting that iNKT cells are crucial for DCs to confer protective Th1/Tc1 immunity. Overall, these data provided direct evidence on the role of iNKT cells in modulating DC function thereby enhancing protective immunity in an *in vivo* model of infection. Since DCs demonstrate a high degree of heterogeneity consisting of various subsets, we further investigated whether this modulating effect of iNKT cells was biased to a DC subset. CD8α+ and CD8α− DCs are important DC subsets residing in the lymphoid tissues such as the spleen. While CD8α+ DCs induce Th1 responses, CD8α− DCs skew Th2 responses (64). These DC subsets were purified from Jα18-KO and WT mice following chlamydial infection and then adoptively transferred to naïve recipient mice that subsequently received chlamydial infection. While both the groups of mice receiving CD8α+ and CD8α− DCs from WT mice showed significant resistance to infection compared to those from Jα18-KO mice, the WT CD8α+ DC recipients had superior protection (63). Collectively, these data provided the first direct evidence that iNKT cells preferentially promote the functional development of a subset of DC to generate protective immunity against infections. Since the local pulmonary immune responses may not be similar to that in the splenic environment, we also examined the iNKT cell–DC interaction in the lung, which is the primary site of infection where predominant inflammatory and immunologic changes occur (65). These findings, however, were not different from what we observed in splenic DC studies, which suggested that DCs residing in different anatomical compartments induce similar immune responses during chlamydial lung infection. Apart from DCs, alveolar macrophages (AMs) are also a critical immune cell population in the lung that regulates immune responses against pulmonary pathogens. In *C. muridarum* infection, iNKT cells were found to affect both the phenotype as well as function of AMs (unpublished observation). Altogether, these findings suggest that iNKT cells exert protective T-cell immunity to *C. pneumoniae* through modulating the function of APCs. How do iNKT cells modulate the DC function? In *C. pneumoniae* infection, the expression of CD40L and IFN-γ by iNKT cells was found to be upregulated (62). To directly examine the contribution of CD40L and IFN-γ in the modulating effect of iNKT cells on DC, we cocultured iNKT cells with DCs and then used blocking antibodies against these molecules. Blockade of either CD40L or IFN-γ significantly reduced the enhancing effect of iNKT cells on IL-12 production by DCs. However, the enhanced effect on IL-12 production was completely abrogated when physical contact between these cells was prevented (62). These data conclude that CD40–CD40L interaction, IFN-γ production, and cell-to-cell contact are critical for iNKT cells to modulate DC function during chlamydial infection.

Recent reports indicate a modulating effect of NKT cells on the function of NK cells. *In vivo* administration of α-GalCer in mice induced NK cells to produce IFN-γ as well as cause cytotoxicity (66, 67). Since NKT and NK cells have been shown to contribute to immunity against chlamydial infections, we focused on whether NKT cells influence the functional role of NK cells during infection (68). We found a reduced expansion of NK cells in Jα18-KO mice following *C. muridarum* infection. A lower percentage of IFN-γ-producing, but higher CD107a+ degranulating, NK cells were observed in Jα18-KO than in WT mice. These data suggest that iNKT cells have a differential effect on NK cell functions. They enhance IFN-γ production by NK cells but inhibit their cytotoxic activities during chlamydial infection (68). Whether the interaction between iNKT and NK cells shape the adaptive immunity merits further exploration.

## iNKT Cells in Chlamydial Pathology

Although a growing wealth of evidence indicates a protective role for iNKT cells in chlamydial infections, some studies have implicated them in eliciting pathologic responses (53, 54, 69). *In vivo* stimulation of iNKT cells by α-GalCer increased *C. muridarum* burden in the lungs of BALB/c mice (69). However, following *C. muridarum* lung infection, CD1d-KO mice (BALB/c background) displayed reduced body weight loss, lung pathology, and chlamydial growth compared to WT mice, which suggested that NKT cells induce immunopathology to *C. muridarum* (69). Similar pathogenic effects of NKT-cell activation were recorded in response to murine genital tract infection with *C. muridarum* (53). When challenged with genital *C. muridarum* infection, WT mice exhibited severe pathologic changes such as oviduct dilation and fibrosis compared to the CD1d-KO mice (53). Altogether these findings suggested a promoting effect of NKT, including iNKT cell, activation on *C. muridarum* infection, which is in contrast to their protective role in *C. pneumoniae* infection (54). The reason behind why iNKT cells act differently in the outcome of infection with these two chlamydial species is still unclear and so warrants further investigation. It appeared that while both pathogens share many biological features, the cellular response and immunological course during infection are different in addition to the differences in growth characteristics and host specificity of these two pathogens. In broad sense, these results suggest that NKT-cell activation effect or the activation itself is pathogen or even species specific. Since *Chlamydiae* may have various antigens for iNKT cells, it is possible that the antigenic variability among the antigens from different chlamydial strains might explain the differential iNKT-cell responses. It is also possible that NKT cells including iNKT cells can play variable roles in different conditions or stages of infection, same as other cell types like CD4 and CD8 T cells. On the other hand, the detrimental role of NKT cells in chlamydial infection has been mostly reported in the studies using CD1d-KO mice that are in BALB/c background and have deficiency in both iNKT and type II NKT cells. Therefore, the observed distinction between chlamydial strains should be more carefully studied. In addition, in-depth studies on the characterization of glycolipid antigens derived from different chlamydial species and analyses of iNKT-cell activation effects in different routes and stages/conditions of infection would provide more insight into the iNKT-cell-mediated pathologic mechanisms in chlamydial infection.

## NKT-Cell Subsets in Chlamydial Infections

Invariant NKT and type II NKT cells have distinct characteristics for their phenotype and function. Whether these cell subsets induce differential immune responses to chlamydial infections is not fully understood. To elucidate the specific roles of iNKT and type II cells in anti-chlamydial immunity, we used CD1d-KO and Jα18-KO mice because the former lack both iNKT and type II NKT cells but the latter are deficient in only iNKT cells. We found that CD1d-KO, in contrast to WT, mice showed increased resistance to *C. muridarum* lung infection. Similar outcome of *C. muridarum* infection was found in case of Jα18-KO. These findings indicate a detrimental role for both iNKT and type II NKT cells in *C. muridarum* infection (54, 69). On the contrary, CD1d-KO and Jα18-KO mice displayed increased susceptibility to *C. pneumoniae* lung infection compared to their respective WT control mice (54), which advocates that NKT-cell subsets induce protection. Taking account of these data, it appears that the protective or pathogenic roles of NKT-cell subsets are mainly driven by the type of bacterial species used to infect mice. Of note, CD1d-KO and Jα18-KO mice used in these studies were having BALB/c and C57BL/6 genetic backgrounds, respectively. Since the outcome of chlamydial infection might be impacted by genetic differences, it would be prudent to use different KO mice with similar genetic background to arrive at a definitive conclusion.

In contrast to the immune function of NKT-cell subsets in chlamydial infections, iNKT and type II NKT cells have been shown to have differential impact on the outcome in certain other models, especially for anti-tumor immunity. *In vitro* stimulation of murine and human iNKT cells with α-GalCer led to an enhanced lysis of tumor cells in a perforin- and granzyme B-dependent fashion, which suggested a direct protective role of these cells in tumor lysis (70, 71). Using a methylcholanthrene (MCA)-induced fibrosarcoma mouse model, Smyth et al. showed that Jα18-KO mice treated with different doses of MCA developed fibrosarcoma, while the control B6 mice did not develop tumors (72). Upon adoptive transfer of the liver lymphocytes from WT mice, Jα18-KO mice, when injected with MCA, exhibited enhanced protection against tumor growth compared to the Jα18-KO mice that received either the liver lymphocytes from Jα18-KO mice or PBS (73). These findings indicate a clear role for iNKT cells in protective immunity to tumor development. In contrast to the anti-tumor activities of iNKT cells, type II NKT cells are reported to suppress the tumor immunosurveillance (74, 75). CD1d-KO, in contrast to Jα18-KO and WT, mice promoted the growth of subcutaneous 15-12RM fibrosarcoma and CT26-L5 colon carcinoma, which indicates that type II NKT cells inhibit the tumor immunosurveillance (74). Similarly, type II NKT cells were suppressive in the immune responses to B-cell lymphomas (75). Overall, these data point out that iNKT cells confer protective immunity to tumors, whereas type II NKT cells promote pathology. In a broader perspective, the data from chlamydial infection and tumor models shed significant light on different roles played by iNKT and type II NKT cells in diverse disease settings. This emphasizes the careful analysis of the impact of NKT-cell subsets on the outcome of diseases because findings from one experimental model cannot be extrapolated to another.

## Conclusion and Future Directions

Invariant NKT cells play an important role in immunity to chlamydial infections. These cells not only induce innate responses but also shape adaptive responses, bridging innate and adaptive immunity. In doing so, iNKT cells modulate the function of DCs through enhanced cytokine production, CD40–CD40L binding, iTCR–antigen interaction, and cell-to-cell contact. In addition, iNKT cells can modulate the function of NK cells that can also modulate DC function. While significant insights have been provided toward understanding the iNKT-cell biology in chlamydial infections, the following questions need to be addressed in the times to come.

(1) The involvement of iNKT cells in both protective immunity and pathology in chlamydial infections has been reported. Notably, the protective and detrimental role of NKT cells in chlamydial infection was mostly shown in Jα18-KO and CD1d-KO mice, respectively. Considering the differences of the mice in genetic background (C57BL/6 vs. BALB/c) and NKT cells (iNKT only vs. iNKT and type II NKT cells), a more detailed study to exclude the influences of these variations need to be performed.
(2) The molecular basis for the influence of iNKT cells on spleen and lung DCs need to be studied in-depth. In addition, it would be interesting to see if there is any impact of iNKT cells on the migration pattern of DC/DC subsets, which is crucial for priming T cells in the lymphoid tissues.
(3) The impact of iNKT cells on other immune cells, apart from NK cells and DCs, merits further investigation. For example, AMs are considered to be a critical immune cell population in pulmonary pathogen defense. How do iNKT cells modulate AMs to influence the outcome of chlamydial infection?
(4) How to prophylactically and therapeutically target iNKT cells for inducing protection without having any significant side effects? Can chlamydial lipid antigens be used in vaccine design strategies for promoting DC function?

## Conflict of Interest Statement

The authors declare that the research was conducted in the absence of any commercial or financial relationships that could be construed as a potential conflict of interest.

## Abbreviations

Ag, antigen; C, *Chlamydia*; CD40L, CD40 ligand; DC, dendritic cell; IC, infected cell; IL-12, interleukin-12; iNKT, invariant natural killer T cell; iTCR, invariant T cell receptor; Tc, CD8+ T cytotoxic cell; Th1, T helper cell.
